# Prognostic Analysis of Postoperative Survival for Ruptured Hepatocellular Carcinoma with or without Cirrhosis

**DOI:** 10.1155/2022/7531452

**Published:** 2022-03-17

**Authors:** Feng Xia, Peng Zhu, Xiao-Ping Chen, Bi-Xiang Zhang, Ming-Yu Zhang

**Affiliations:** ^1^Department of Hepatic Surgery Center, Tongji Hospital of Tongji Medical College, Huazhong University of Science and Technology, Wuhan, Hubei, China; ^2^Department of Digestive Medicine, Tongji Hospital of Tongji Medical College, Huazhong University of Science and Technology, Wuhan, Hubei, China

## Abstract

**Background and Aims:**

Conflicting results are often observed in the prognosis of patients with ruptured hepatocellular carcinoma (rHCC), and there are currently very few studies on the long-term postoperative outcomes of ruptured hepatocellular carcinoma patients. This study aimed to distinguish between the postoperative prognosis of rHCC patients with cirrhosis (rHCC-C) and those without cirrhosis (rHCC-NC) using some serum markers.

**Methods:**

We collected the data of 151 rHCC patients treated at our centers from January 2010 to March 2021. 62 had no cirrhosis, and 89 had cirrhosis. The prognosis of rHCC-C and rHCC-NC groups was compared using the Kaplan-Meier method. We used multivariate Cox regression to analyze prognostic factors in rHCC patients, and subgroup analysis was performed on the two groups of patients.

**Results:**

The long-term prognosis of rHCC-NC patients was better than that of rHCC-C patients. Tumor diameter, Barcelona clinic liver cancer (BCLC) stage, HBsAg, positive Hepatitis C virus (HCV) antibodies, elevated creatinine, and elevated T-bilirubin were prognostic factors for overall survival (OS) in rHCC-C patients. However, only alpha-fetoprotein (AFP) > 92 ng/mL was a prognostic factor for OS in rHCC-NC patients. In noncirrhotic patients, HBsAg positivity was only associated with OS. Similarly, the presence or absence of microvascular invasion (MVI) also had different results in the two groups.

**Conclusions:**

There are differences in serum alpha-fetoprotein (AFP) levels, the presence of microvascular invasion (MVI), and HBsAg positivity between rHCC-C and rHCC-NC patients, indicating that the analysis of these prognostic factors may help improve the management of rHCC patients and provide a direction for future treatment options.

## 1. Introduction

The annual number of new cases of hepatocellular carcinoma (HCC) worldwide is approximately 800,000, and it is the third most common cause of cancer death. Simultaneous rupture is a complication of HCC, and its prognosis is poor [1–3]. The proportion of spontaneous rupture of HCC varies among regions and is higher in Asia and Africa. In Asia, 10% of annual deaths because of HCC are in patients with ruptured HCC. According to certain studies, the acute phase of rHCC leads to a mortality rate as high as 25% to 75% [4, 5].

Cirrhosis is the basis of HCC in 70% to 80% of the cases worldwide [6, 7]. Almost all patients who develop liver cancer from cirrhosis have chronic necrosis, which shows that chronic necrotizing inflammation plays a vital role in the development of HCC. The currently known etiologies of HCC are mainly HBV or HCV infection, and a history of alcohol consumption. In the Western world, it has been found that almost all HCC patients have a history of HBV, HCV, or alcohol consumption [8–10]. In Asian countries, it is mainly associated with HBV infection. However, the proportion of HBsAg positivity in cirrhotic and noncirrhotic rHCC patients is not clear.

It is essential to identify some tumor-related factors and markers to predict the postoperative prognosis of patients with rHCC. Among the reported factors associated with postoperative prognosis of rHCC, tumor length ≥10 cm and lack of tumor encapsulation were the only independent prognostic factors for poor OS and recurrence-free survival (RFS). However, the role of other factors, such as age, gender, and preoperative serum alpha-fetoprotein has not been discussed in previous studies [11–13]. Unfortunately, although many studies have shown that cirrhosis is an important factor in the process of tumorigenesis, we found that in previous prognostic studies, investigators only studied rHCC patients with cirrhosis alone [14–17] or rHCC patients without cirrhosis alone [18] after comprehensively searching the available literature. In our study, we first grouped the patients with rHCC according to the presence or absence of cirrhosis and then performed a subgroup analysis of patients with or without cirrhosis.

Usually, researchers will select certain disease biomarkers or pathological factors to predict the prognosis of patients with rHCC. Alpha-fetoprotein (AFP) is a glycoprotein that belongs to the albumin family. It is a significant tumor marker. At present, AFP is mainly used as a serum marker for the diagnosis and treatment efficacy monitoring of primary liver cancer in clinical practice [19, 20]. High AFP is associated with poor prognosis in patients with many solid tumors. However, the cutoff point and the prognostic value of AFP in patients with rHCC are uncertain [21, 22]. Microvascular invasion (MVI) status has been reported to help clinicians determine treatment plans. The presence or absence of MVI is usually reported in postoperative pathology, and when MVI is positive, it indicates that the patient's prognosis may be poor and there is a risk of recurrence [23]. Some investigators use certain indicators to predict MVI preoperatively to manage the patient better [24–26]. In patients with rHCC, it is also an important factor in predicting prognosis, however, no study has analyzed the predictive value of MVI between cirrhosis and noncirrhosis groups [23, 26–28]. There are contradictions in the reported effect of positive or negative HBsAg on HCC, with most studies suggesting that positive HBsAg is an independent factor for a poor prognosis of HCC. In contrast, some studies suggest that HBsAg positivity has no significant impact on the prognosis of HCC patients [29–32]. According to some recent guidelines, antiviral therapy is still necessary for HBsAg-positive patients [33]. At present, it is still controversial whether positive serum HBsAg affects the prognosis of HCC patients.

This study assessed clinicopathological and prognostic differences in ruptured HCC patients with or without cirrhosis. We retrospectively collected the information of 151 patients who had hepatectomy for rHCC at our institution during a period of 11 years. All postoperative rHCC patients were divided into cirrhosis and noncirrhosis groups, and the prognoses of the two groups were analyzed. The effects of AFP, MVI, and HBsAg on rHCC recurrence-free survival and overall survival were assessed in rHCC-C and rHCC-NC. Our present hypothesis is that there is a difference between these two groups.

## 2. Material and Methods

### 2.1. Patients

**Figure 1:**
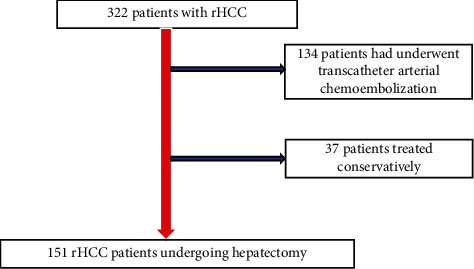
Flow chart about patients' selection.

Data for 151 patients who had hepatectomy in our hospital from January 2010 to March 2021 because of the spontaneous rupture of hepatocellular carcinoma were extracted from the Department of Hepatic Surgery, Tongji Hospital, Wuhan (Figure 1). Experienced liver surgeons performed all liver procedures. The variables obtained included patient gender, age, longest tumor diameter, the number of tumors, the presence of portal hypertension, the presence of tumor microvascular invasion, BCLC stage of the tumor, the pathological differentiation of tumor, preoperative AFP, preoperative ALP, preoperative AST, preoperative ALT, and presence of necrosis. Experienced radiologists determined tumor rupture using enhanced CT scanning and abdominal MRI. Experienced pathologists confirmed the HCC diagnosis in our hospital. We defined the inclusion criteria for patients as follows: [1] pathologically confirmed hepatocellular carcinoma [2], single tumor, and [3] Child-Pugh grade A or B. The exclusion criteria were as follows: [1] the pathological diagnosis was not HCC [2], positive surgical margins [3], presence of lymph node metastasis [4], macrovascular invasion [5], patients with recurrence and reresection, and [7] incomplete follow-up information and clinical data. Our research was authorized by the Ethics Committee of Wuhan Tongji Hospital (TJ-IRB20210205), and patients who signed the informed consent form were included in the study (Figure 1).

### 2.2. Definitions

**Figure 2:**
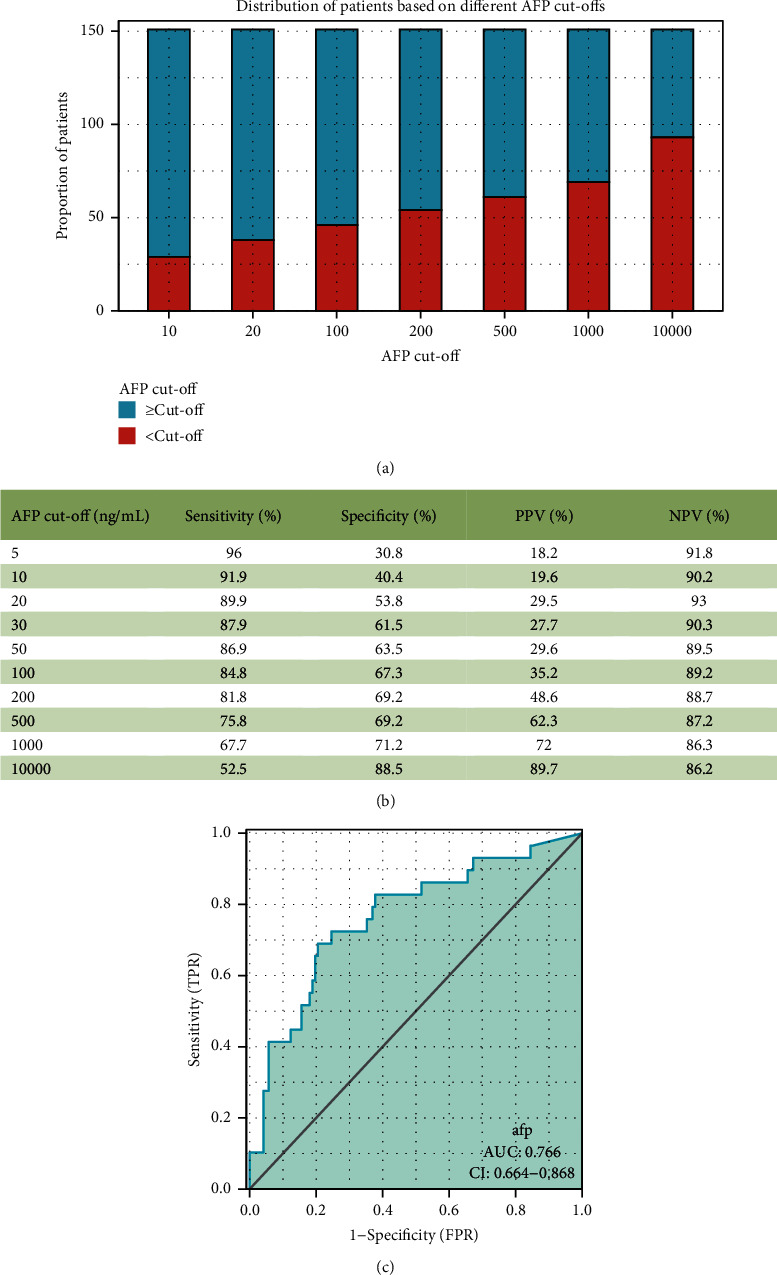
(a) Distribution of patients based on different AFP cut-offs, presented as a histogram. AFP: alpha-fetoprotein. (b) Sensitivity, specificity, positive predictive value (PPV), and negative predictive value (NPV) for predicting HCC recurrence after ruptured liver cancer surgery based on various AFP cut-off values. The area under the receiver operating characteristic (ROC) curve (AUC) of (c). AFP for predicting HCC recurrence after ruptured liver cancer surgery.

Cirrhosis is a common chronic progressive liver disease formed by the repeated action of one or more etiologies on the liver. Preoperative abdominal ultrasonography was performed to assess the presence of cirrhosis in the patients included in this study, and two experienced pathologists determined the presence of cirrhosis based on the pathological characteristics of postoperative liver specimens. Ruptured hepatocellular carcinoma surgery is generally completed one week after admission, and the levels of various serum parameters are measured at admission. For the optimal cutoff value of AFP, the receiver operating characteristic curve (ROC) curve for predicting recurrence after hepatectomy was drawn, and the sensitivity and specificity were calculated. Finally, the Youden index was calculated to find the corresponding value with the largest Youden index, AFP = 92 ng/mL(Figure 2).

### 2.3. Follow-Up

According to the European Association for the Study of the Liver (EASL) guidelines (33), we administer antiviral therapy (antiviral therapy mainly includes two types: subcutaneous PegIFN*α*, or oral entecavir (ETV), tenofovir disoproxil fumarate (TDF), and tenofovir alafenamide (TAF)). All patients with rHCC had follow-up visits every quarter in the first year and every half year in the second year after the operation. During each follow-up, the liver and kidney functions, routine blood tests, blood biochemistry, and medical imaging, including abdominal enhanced CT, were performed to determine tumor recurrence. If the possibility of recurrence was suspected, further abdominal MRI, and sometimes, PET-CT would be performed. For patients with recurrence, retreatment options include surgical reresection, transcatheter arterial chemoembolization (TACE), and radiofrequency ablation (RFA). The overall survival (OS) was defined as the time interval from the first day after surgery to the date of death or last follow-up. Recurrence-free survival (RFS) was defined as the time interval from the first day after surgery to the discovery of a neoplasm in the liver or other sites, or the date of the last follow-up for those without recurrence.

### 2.4. Data Analysis and Expression

Continuous data satisfying a normal distribution were expressed as mean ± standard deviation (*M* ± SD) and compared using the independent sample *t*-test. Continuous data that were not normally distributed were expressed using the median sum (25% and 75%) (Q1–Q3) and compared by the Mann–Whitney test. Categorical data were expressed as numbers and proportions and compared by the chi-squared test, Yates correction, or Fisher's exact test. Cumulative OS and RFS rates were estimated by plotting survival curves using the Kaplan–Meier method. OS/RFS (overall survival or recurrence-free rate), expressed as *S* (*tk*), refers to the probability of survival or nonrecurrence after *tk* unit time. If there is no deletion data, it refers to the number of cases still alive at *Tk* moment/the total number of cases at the beginning of observation. If there is missing data, the denominator needs to be corrected on time, and the K-M method for survival rate at this time is calculated as *S* (*tk*) = *P* (*T* > *tk*) = *p*1 ∗ *p*2 ∗ … *p*k, and the curves were compared using the log-rank test. Multivariate cox regression models were performed to identify the predictors of OS and RFS using the forward method. Significant variables (*p* < 0.1) in the univariate analysis were included in the Cox model. In this study, ROC was used to compare different variables' predictive discrimination and clinical utility.

Kaplan–Meier or multivariate analyses were performed using SPSS 25.0 software (IBM, Armonk, New York, USA) and R software (version 4.0.5, version 4.0.5, R Foundation for Statistical Computing, Vienna, Austria). *P* values <0.05 (both sides) were considered statistically significant. The calculation of sample size had been performed by PASS (Version: 11.0) before the study was conducted.

## 3. Results

### 3.1. Patient Characteristics

151 patients with ruptured HCC were included in our study (Figure 1). The demographic and essential characteristics of the patients are shown in Table 1. Among them, 62 patients (41.1%) did not have cirrhosis, and 59 patients (58.9%) had cirrhosis. There were 133 males (88.1%) and 18 females (11.9%), and the mean age was 46.4 ± 11.7 years. Among all patients, 129 (85.4%) had no MVI, and 22 (14.6%) had MVI. 125 (82.8%) were HBsAg-positive, and 26 (17.2%) were negative. Among the patients with liver cirrhosis, 72 (80.9%) had no MVI, and 17 (19.1%) had MVI. 87 (97.8%) were HBsAg-positive, and 2 (2.2%) were HBsAg-negative. Among the patients without liver cirrhosis, 57 (91.1%) had no MVI, and 5 (8.1%) had MVI. 38 (61.3%) were HBsAg-positive, and 24 (38.7%) were HBsAg-negative. The average tumor diameter for the entire cohort was 7.8 cm (5.2–10.2) (Table 1).

### 3.2. Liver Cirrhosis Affected the Long-Term HCC Patient Prognosis

**Figure 3:**
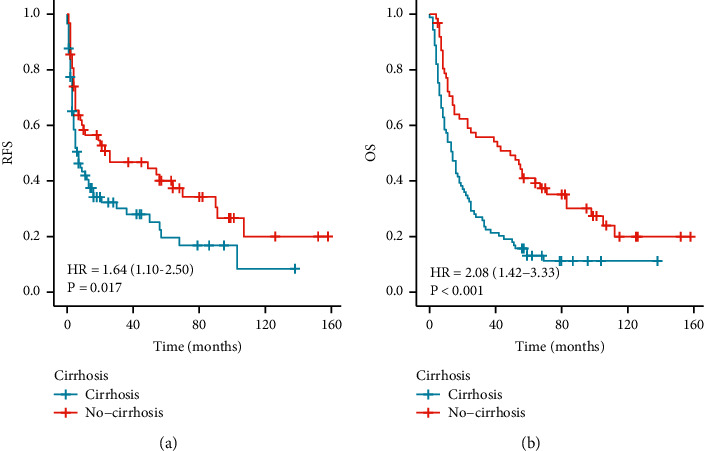
RFS (a) and OS (b) in all patients with or without cirrhosis.

Cirrhosis affected the long-term prognosis in patients with rHCC, and the median (interquartile range [IQR] (25%–75%)) and mean (interquartile range [IQR] (25%–75%)) RFS at follow-up were 10 (3.46–16.54) months and 44.42 (33.50–55.34) months, respectively, in all patients. The median and mean RFS times were 26.0 and 57.2 months in rHCC-NC patients and 7.0 and 31.7 months in rHCC-C patients (*P* = 0.015) (Figure 3(a)). The 1-, 3-, and 5-year RFS rates were 51.0%, 46.8%, and 21.3% for HCC-NC patients and 33.0%, 28.1%, 11.1% for rHCC-C patients, respectively (*P* < 0.05, Figure 3(a)). The 1-, 3-, and 5-year OS rates were 34.0%, 19.2%, and 10.1% for HCC-C patients and 59.0%, 51.2%, and 20.2% for rHCC-NC patients, respectively. The median and mean OS times were 49.0 and 62.9 months for rHCC-NC patients and 14.0 and 30.7 months for rHCC-C patients. In summary, our results indicate that cirrhosis is one of the prognostic factors for poor long-term outcomes after hepatectomy in patients with rHCC (Figure 3(b)).

Patients without cirrhosis had a hazard ratio of 2.08 (95% confidence interval 1.42–3.33; *P* < 0.001) for OS and 1.64 (95% confidence interval 1.10–2.50; *P* = 0.017) for RFS (Figures 3(a) and 3(b)).

### 3.3. Prognostic Factors for Survival in rHCC Patients

Multivariate regression showed that MVI and AFP were independent predictors of RFS, and tumor length, BCLC stage, HBsAg, and HCV were independent predictors of OS in all rHCC patients with cirrhosis. AFP and D-bilirubin were independent predictors of RFS, and AFP was an independent predictor of OS in all rHCC patients without cirrhosis.

Multivariate analysis was performed for rHCC-C and rHCC-NC. MVI and AFP >92 ng/mL were associated with poor RFS in rHCC-C patients, and AFP >92 ng/mL and high Dbilirubin were associated with poor RFS in rHCC-NC. Long tumor diameter, poor BCLC stage, positive HBsAg, HCV, elevated creatinine, and high T-bilirubin were associated with poor OS in rHCC-C, and AFP >92 ng/mL was associated with poor OS in rHCC-C. In addition, we observed that the HBV-DNA load was much more significant in the cirrhotic group than in the noncirrhotic group (Tables 2–5).

### 3.4. Disease Markers and Prognosis in rHCC Patients

**Figure 4:**
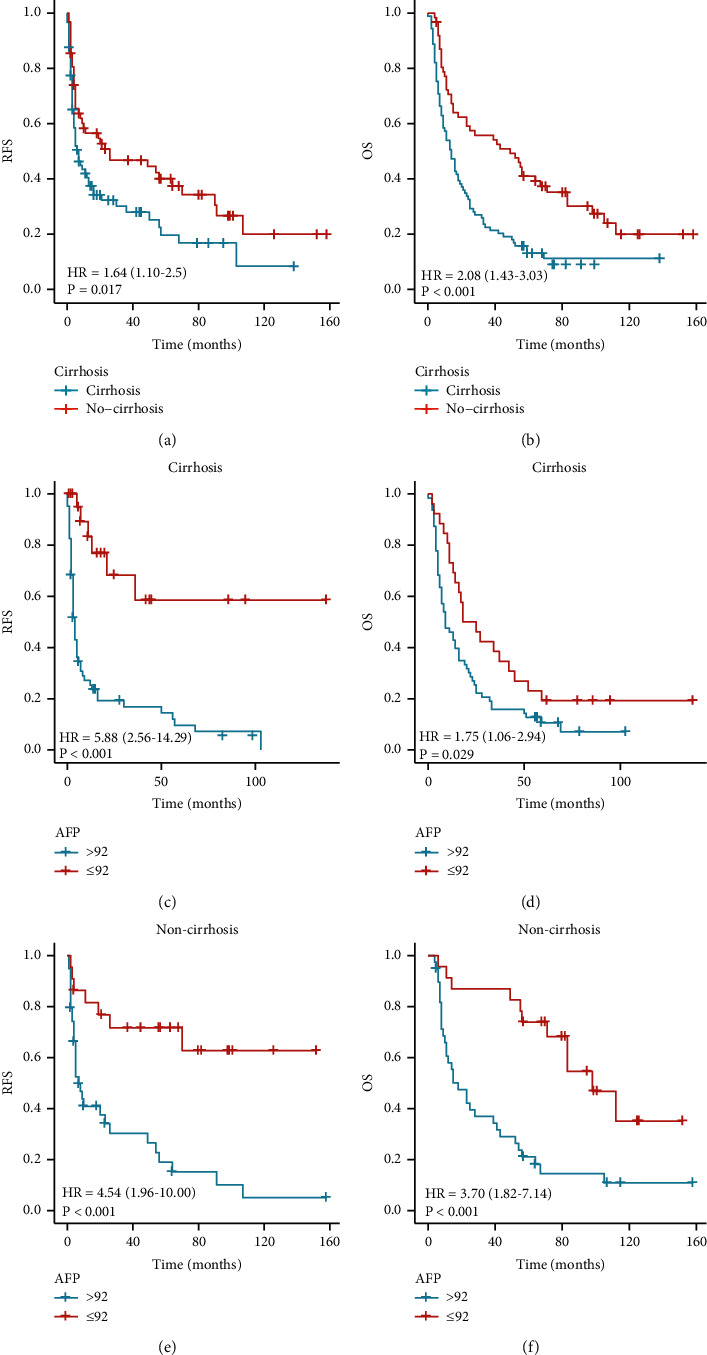
(a, b) RFS and OS of all rHCC patients with different AFP levels, (c, d) RFS and OS of rHCC-C patients with different AFP levels, and (e, f) RFS and OS of rHCC-NC with different AFP levels.

**Figure 5:**
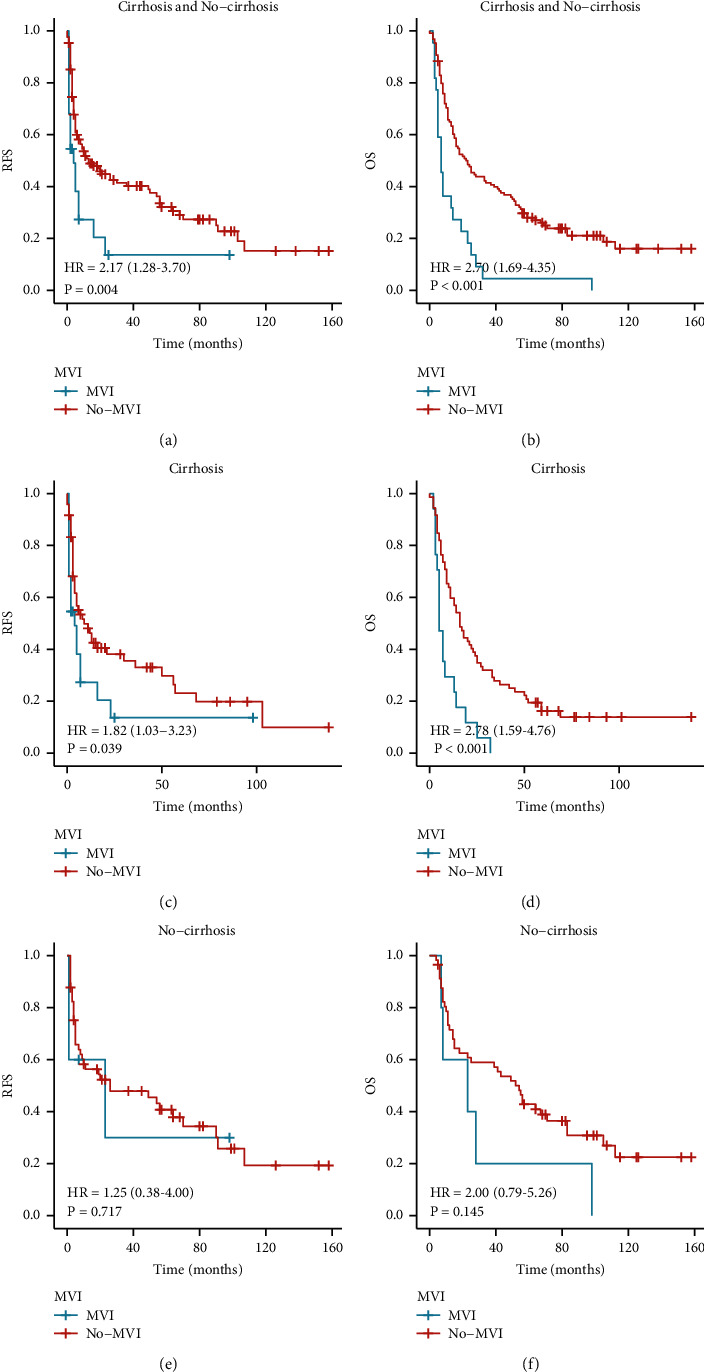
(a, b) RFS and OS of all rHCC patients with or without Microvascular invasion (MVI), (c, d) RFS and OS of rHCC-C patients with or without MVI, and (e, f) RFS and OS of rHCC-NC patients with or without MVI.

**Figure 6:**
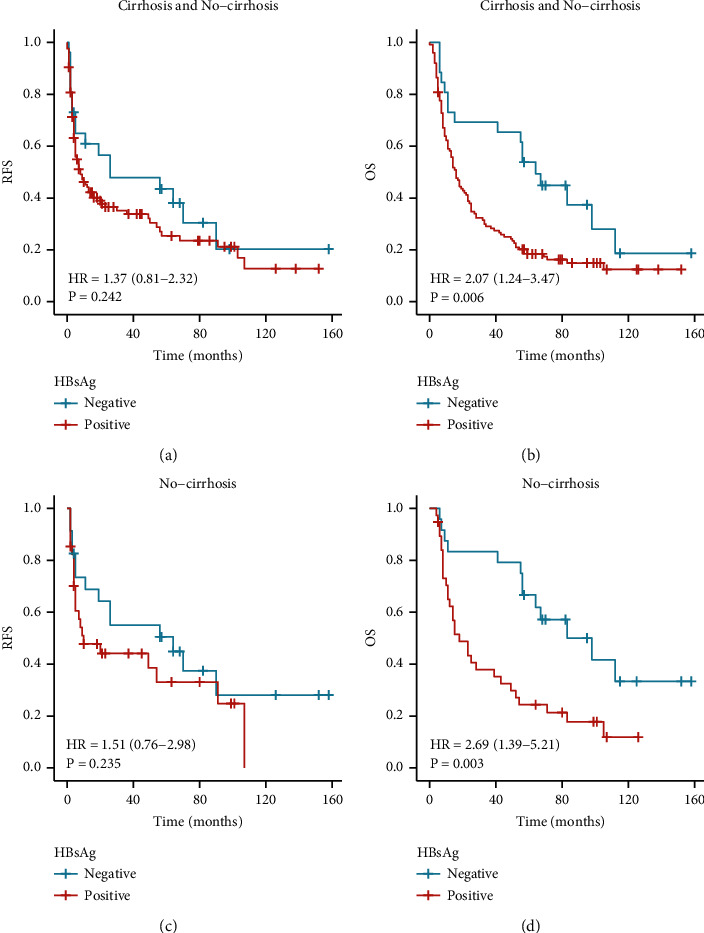
(a, b) RFS and OS of all patients with HbsAg(±), (c, d) RFS and OS of rHCC-NC patients with HbsAg(±).

We also compared the clinical value of AFP, MVI, and HBsAg in predicting the prognosis of the rHCC-NC group. We found that all three were linked to RFS and OS in rHCC patients (Figures 4(a) and 4(b); Figures 5(a) and 5(b); Figures 6(a) and 6(b)). To further analyze the value of AFP in rHCC-C and rHCC-NC, we compared AFP >92 ng/ml with AFP <92 ng/ml, and the AFP value was associated with RFS and OS in both groups (Figures 4(c), 4(d), 4(e), and 4(f)).

In all patients, the median and mean RFS were 14.0 and 98.6 months for patients with AFP ≤92 ng/mL. They were 5.0 and 23.1 months for patients with AFP >92 ng/mL, 55.0 and 70.1 months for patients with AFP ≤92 ng/mL, and 13.0 and 31.0 months for patients with AFP >92 ng/mL. For patients with rHCC-C, the median and mean RFS were 10.0 and 88.2 months for patients with AFP ≤92 ng/ml, 4.0 and 17.1 months for patients with AFP >92 ng/ml, 18.0 and 44.5 months for patients with AFP ≤92 ng/ml, and 9.0 and 21.9 months for patients with AFP >92 ng/ml.

For patients with rHCC-NC, the median and mean RFS were 40.0 and 100.6 months for patients with AFP ≤92 ng/ml, 7.0 and 31.4 months for patients with AFP >92 ng/ml, 98.0 and 97.7 months for patients with AFP ≤92 ng/ml, and 18.0 and 40.4 months for patients with AFP >92 ng/ml.(Figures 4(a)–4(f)).

All tumor samples were pathologically examined after the operations. A total of 22 tumor samples were found to have MVI, 17 (19.1%) in tumor samples with cirrhosis, and 5 (8.1%) in tumor samples without cirrhosis. From figures 5(a)–5(d), it can be seen that MVI was correlated with RFS and OS in all patients and patients with cirrhosis. From figures 5(e), 5(f), it can be seen that MVI was not correlated with RFS and OS in rHCC patients without cirrhosis (*P* = 0.717, *P* = 0.145) (Figure 5).

A total of 125 patients had positive serum HBsAg after hepatitis B surface antigen confirmatory testing, of which 87 patients had liver cirrhosis, making up 97.8% of the cirrhotic group. 38 did not have liver cirrhosis, making up 61.3% of the noncirrhotic group. Since only two (2.2%) were HBsAg-negative in rHCC-C patients, RFS and OS could not be analyzed in rHCC-C patients.

In the analysis of all patients, HBsAg positivity did not correlate with the RFS of patients (*P* = 0.242), however, it was associated with a worse prognosis (*P* = 0.006) in terms of the OS (figures 6(a) and 6(b)). In patients without cirrhosis, HBsAg positivity and negativity were similarly not correlated with the patients' RFS (*P* = 0.235) but correlated with the patients' OS (*P* = 0.003). Positive HBsAg indicated a worse prognosis (figures 6(c) and 6(d)).

## 4. Discussion

Rupture is a severe complication of liver tumors, and it generally has an inferior prognosis. The proportion of ruptured HCC has been increasing yearly, reaching 10% to 15% in some parts of Asia. With a reported mortality rate as high as 32% once rupture occurs [4], it deserves our attention. At present, the surveillance of rHCC has been greatly improved, and tumor rupture can be detected very early from the symptoms and imaging studies. It is important to identify markers for predicting the prognosis of patients with resectable rHCC after tumor resection. Among the previously reported related factors of rHCC or HCC, the characteristics of the tumor itself, such as tumor size, tumor number, etc., are commonly used to determine OS and RFS, and some other factors, such as pathological factors, tumor markers, etc., are also considered to affect the prognosis of patients. Unfortunately, although cirrhosis is a commonly known cause of liver tumorigenesis, in previous studies, the investigators only discussed rHCC with cirrhosis or rHCC without cirrhosis [14–17] and did not categorize patients into rHCC-C and rHCC-NC for subgroup prognosis analysis. Our study evaluated the effects of tumor markers, pathological factors, and hepatitis B antigen on the survival and recurrence of rHCC in both groups.

In the Asian region, almost all cases of HCC developed from cirrhosis are caused by chronic hepatitis B virus infections. According to existing studies, most HCC is gradually developed in patients with cirrhosis, however, if HCC patients do not have cirrhosis, it is considered to be de novo [6]. In the published literature, the prognostic value of HBsAg is different in different studies. Janssen et al. and Hu L [34, 35] believed that HBsAg has little effect on the prognosis, while Sohn et al. [32] believed that both HBsAg and HBV DNA are important risk factors for early and late recurrence in HBV-related HCC patients after surgery. This article is the first such one in which the prognostic value of HBsAg has been analyzed in rHCC patients, stratified based on cirrhosis, and we got different results in different subgroups. It can be seen from our baseline data table that in patients with cirrhosis, up to 97.8% of patients were HBsAg positive, while in patients without cirrhosis, only 61.3% were HBsAg positive. In our study, HBsAg positivity did not affect the postoperative RFS in rHCC patients without cirrhosis, however, it affected the postoperative OS. Hence, antiviral therapy was also necessary for patients, and the recent EASL guidelines [33] also recommend antiviral therapy for HBV-related HCC patients. Notably, we found that only in noncirrhotic patients, multivariate analysis revealed that a higher hepatitis B viral load was associated with worse recurrence-free survival. At the same time, we observed that the viral load was much higher in patients with postoperative recurrence. Therefore, antiviral therapy may have a role in preventing the recurrence of rHCC after surgery [32, 36–39]. Furthermore, it is necessary to discuss the effect of HBV-DNA viral load on OS and RFS. Previous studies have shown that antiviral therapy can inhibit viral load elevation and inhibit viral replication in hepatocytes, which can give patients an opportunity to receive further treatment, and inhibiting viral load elevation can improve prognosis. Sohn et al. [32] found that the viral load was associated with early recurrence, which was somewhat different from the results of our study. Qu et al. [40] further defined the viral load, and they believed that HBsAg >250 IU/mL at the time of tumor resection was an independent risk factor for late recurrence. Yan et al. [41] believed that serum HBV DNA levels should be measured at multiple time points, and viral suppression to low levels was beneficial to patients.

At present, certain tumor markers are being used by researchers to predict the prognosis of rHCC patients after surgery. AFP is the most classical tumor marker, and 400 ng/mL is often used as the cutoff value in patients with nonruptured HCC. However, different studies have a different selection of the cut-off value of AFP in rHCC [42–45]. Hence, we chose to use an ROC curve to determine the cutoff value, and we evaluated the relationship between AFP and the OS and RFS in rHCC patients with or without cirrhosis. This study found that high preoperative serum AFP levels were associated with a worse prognosis regardless of the presence or absence of cirrhosis in patients with rHCC. It is consistent with the conclusions of other studies. Zhang et al. [13] suggested that AFP ≥1000 ng/mL is an independent risk factor affecting 30-day mortality and a prognostic factor for OS in rHCC. Kerdsuknirun et al. [44] set the cut-off to 20 and 200 ng/ml, and they found that AFP levels >200 ng/ml were more common in patients with ruptured HCC. Our study also found that patients with high AFP accounted for more rHCC patients.

MVI has been a hot topic in recent years and a key determinant of early recurrence and survival [23]. However, the presence of MVI is difficult to determine based on preoperative images alone. Yang et al. [26] constructed an MVI prediction model using imaging and radionics, and Lei et al. [46–48] constructed a model for predicting MVI based on some preoperative serological markers. In summary, according to the preoperative prediction, different surgical options and treatment measures are taken, including expanding the extent of resection or stratified management of different risk groups. However, there is no model to predict MVI in rHCC patients. Therefore, further analysis of MVI is necessary, and in this study, we found that among all patients with rHCC, patients with MVI had worse OS or RFS. Among patients with cirrhosis, those who also had MVI had worse OS or RFS performance. However, it should be noted that the presence or absence of MVI did not affect the OS or RFS in patients without cirrhosis. Previous studies concluded that the OS and RFS are worse whenever the postoperative pathology analysis reports the presence of MVI [23, 28, 49, 50]. It may also guide future work on whether there is a greater need to focus on the situation of MVI in rHCC patients with cirrhosis.

This study has several limitations. RHCC cases are rare, and multiple factors influence the prognosis. However, we still set strict inclusion criteria and tried to exclude some confounding factors to better evaluate the prognosis of the cirrhotic and the noncirrhotic groups. The stringent criteria described in our methods significantly reduced the number of qualified rHCC cases to be included in our study. Although the study included 151 patients with HBV-related HCC, the sample size was still not large enough, which reduced the power of the statistical analysis.

In conclusion, this research demonstrates that rHCC-C and rHCC-NC patients differ in their histopathological and clinical characteristics, prognosis, and outcome. Patients with rHCC are in an urgent situation at admission, and they need to be managed promptly. The long-term prognosis of noncirrhotic patients was better than that of cirrhotic patients. AFP >92 ng/mL and positive MVI were associated with a worse prognosis, regardless of whether the patient has cirrhosis, while HBsAg positivity was associated with a poorer prognosis in rHCC patients with cirrhosis, and it did not affect the prognosis of rHCC patients without cirrhosis. Hence, it is essential to group rHCC patients based on whether they have cirrhosis. Stratified analyses based on the presence of cirrhosis can reduce conflicting conclusions regarding the prognosis of patients with rHCC and help guide clinicians to manage patients with rHCC better.

## Figures and Tables

**Table 1:** The clinical data of ruptured HCC patients with cirrhosis or without cirrhosis.

	Total	Without cirrhosis	With cirrhosis	*P* value
Variables (%)	*n* = 151	*n* = 62 (41.1)	*n* = 89 (58.9)	
gender (%)				0.756
Male	133 (88.1)	54 (87.1)	79 (88.8)	
Female	18 (11.9)	8 (12.9)	10 (11.2)	
Age (y)	46.4 ± 11.7	47.0 ± 1.5	45.9 ± 1.2	0.516
Length (cm)	7.8 (5.2–10.2)	7.0 (5.0–10.2)	7.9 (5.9–10.2)	0.295
Number (%)				0.857
Single	118 (78.1)	48 (77.4)	70 (78.7)	
Multiple	33 (21.9)	14 (22.6)	19 (21.3)	
MVI (%)				0.049
No	129 (85.4)	57 (91.1)	72 (80.9)	
Yes	22 (14.6)	5 (8.1)	17 (19.1)	
Regular excision (%)			0.17	
No	113 (74.8)	50 (80.6)	63 (70.8)	
Yes	38 (25.2)	12 (19.4)	26 (29.2)	
BCLC (%)				0.055
A	88 (58.3)	39 (62.9)	49 (55.1)	
B	31 (20.5)	16 (25.8)	15 (16.9)	
C	32 (21.1)	7 (11.3)	25 (28.1)	
Edmondson-Steiner (%)				0.001
1	17 (11.3)	13 (21.0)	4 (4.5)	
2	69 (45.7)	32 (51.6)	37 (41.6)	
3	38 (25.2)	9 (14.5)	29 (32.6)	
4	27 (17.9)	8 (12.9)	19 (21.3)	
AFP (ng/ml)				0.309
≤92	49 (32.5)	23 (37.1)	26 (29.2)	
>92	102 (67.5)	39 (62.9)	63 (70.8)	
HBV-DNA (copies/ml)	7665.0 (578.5–284250.0)	3300.0 (100.0–32325.0)	15275.0 (1565.0–343157.5)	0.04
Necrosis (%)				0.702
No	112 (74.2)	47 (75.8)	65 (73.0)	
Yes	39 (25.8)	15 (24.2)	24 (27.0)	
Local invasion (%)				0.085
No	75 (49.7)	36 (58.1)	39 (43.8)	
Yes	76 (50.3)	26 (41.9)	50 (56.2)	
HBsAg (%)				<0.001
No	26 (17.2)	24 (38.7)	2 (2.2)	
Yes	125 (82.8)	38 (61.3)	87 (97.8)	
HCV (%)				0.362
No	142 (94.0)	57 (91.1)	85 (95.5)	
Yes	9 (6.0)	5 (8.1)	4 (4.5)	
Alcohol history (%)				0.143
No	114 (75.5)	43 (69.4)	71 (79.8)	
Yes	37 (24.5)	19 (30.6)	18 (20.2)	
ALB (g/L)	35.2 ± 5.5	34.64 ± 6.43	35.58 ± 4.72	0.303
ALT (U/L)	29 (21–44)	25.8 (19.8–41.0)	31.0 (24.0–51.0)	0.065
AST (U/L)	37 (25–65)	35.5 (22.8–61.3)	38.0 (29.0–66.0)	0.095
ALP (U/L)	80 (61–91)	80.0 (53.8–53.2)	80.2 (65.0–95.5)	0.078
GGT (U/L)	62 (34.4–91.0)	61.5 (27.5–73.2)	62.0 (109.0–36.0)	0.029
Creatinine (*μ*mol/L)	68.8 (61.0–78.0)	66.5 (58.9–76.0)	70.4 (64.0–79.0)	0.044
Pre-ALB (g/L)	141.1 (129.0–172.0)	144.1 (131.7–180.3)	135.8 (128.3–168.5)	0.159
Dbilirubin (*μ*mol/L)	4.7 (3.5–6.9)	4.4 (3.3–6.9)	5.0 (3.6–6.9)	0.434
Tbilirubin (*μ*mol/L)	14.9 (11.0–20.0)	14.9 (11.0–19.8)	14.7 (11.1–20.7)	0.89
T-cholesterol (mmol/L)	3.5 (3.0–4.2)	3.5 (2.8–4.2)	3.5 (3.0–4.1)	0.959

AFP: alpha-fetoprotein; MVI: microvascular invasion; BCLC: Barcelona clinic liver cancer; HCV: hepatitis C virus; ALB: albumin; ALT: alanine aminotransferase; AST: aspartate aminotransferase; ALP: alkaline phosphatase; GGT: glutamyl transpeptidase; HCC: hepatocellular carcinoma.

**Table 2:** Univariate and multivariate analysis for RFS in rHCC-C.

	Univariate analysis			Multivariate analysis		
	*P*	HR	95% confidence interval	*P*	HR	95% confidence interval
Gender	0.317	0.427	0.081–2.260			
Female/Male						
Age (per y)	0.249	0.981	0.949–1.014			
Length (per cm)	0.118	1.102	0.976–1.245			
Number	0.021	2.909	1.178–7.184			
Multiple/single						
MVI	0.057	3.128	0.967–10.116	0.022	2.077	1.110–3.887
Yes/No						
BCLC	0.357	0.754	0.413–1.375			
C/B/A						
Edmondson	0.110	1.417	0.924–2.175			
IV/III/II/I						
AFP	0.004	4.331	1.617–11.601	<0.001	4.957	2.090–11.758
>92 ng/ml/≤92 ng/ml						
HBV-DNA (per 1)	0.562	1	1.000–1.000			
Necrosis	0.209	0.607	0.278–1.323			
No/Yes						
Local invasion	0.287	1.569	0.685–3.593			
Yes/No						
HbsAg	0.459	0.363	0.025–5.305			
No/Yes						
HCV	0.476	0.51	0.080–3.248			
No/Yes						
Alcohol history	0.948	0.974	0.437–2.170			
No/Yes						
ALB (per g)	0.831	0.99	0.899–1.090			
ALT (per U)	0.229	1.007	0.996–1.017			
AST (per U)	0.322	0.997	0.990–1.003			
ALP (per U)	0.917	1	0.994–1.007			
GGT (per U)	0.105	1.004	0.999–1.008			
Creatinine (per *μ*mol)	0.678	1.006	0.978–1.034			
Pre-ALB (per g)	0.365	1.004	0.995–1.014			
Dbilirubin (per *μ*mol)	0.411	0.909	0.724–1.141			
Tbilirubin (per *μ*mol)	0.727	1.017	0.924–1.120			
Tcholesterol (per mmol)	0.745	0.945	0.670–1.331			

AFP: alpha fetoprotein; MVI: microvascular invasion; BCLC: Barcelona clinic liver cancer; HCV: hepatitis C virus; ALB: albumin; ALT: alanine aminotransferase; AST: aspartate aminotransferase; ALP: alkaline phosphatase; GGT: glutamyl transpeptidase; HCC: hepatocellular carcinoma.

**Table 3:** Univariate and multivariate analysis for RFS in rHCC-NC.

	Univariate analysis			Multivariate analysis		
	*P*	HR	95% confidence interval	*P*	HR	95% confidence interval
Gender	0.657	1.447	0.283–7.401			
Female/Male						
Age (per y)	0.135	1.037	0.989–1.088			
Length (per cm)	0.368	1.086	0.908–1.298			
Number	0.441	1.650	0.461–5.901			
Multiple/Single						
MVI	0.227	0.233	0.022–2.481			
Yes/No						
BCLC	0.420	1.432	0.599–3.425			
C/B/A						
Edmondson	0.398	0.805	0.488–1.330			
IV/III/II/I						
AFP	0.003	5.097	1.709–15.204	<0.001	4.122	1.857–9.146
> 92 ng/ml/≤92 ng/ml						
HBV-DNA (per 1)	0.034	1.000	1.000–1.000			
Necrosis	0.499	0.670	0.210–2.140			
No/Yes						
Local invasion	0.412	0.607	0.184–2.001			
Yes/No						
HbsAg	0.891	0.931	0.336–2.583			
No/Yes						
HCV	0.019	11.018	1.479–82.099			
No/Yes						
Alcohol history	0.389	1.644	0.530–5.102			
No/Yes						
ALB (per g)	0.360	0.967	0.899–1.039			
ALT (per U)	0.145	0.982	0.959–1.006			
AST (per U)	0.998	1.000	0.987–1.013			
ALP (per U)	0.906	0.999	0.980–1.018			
GGT (per U)	0.918	1.001	0.984–1.018			
Creatinine (per *μ*mol)	0.301	0.983	0.952–1.015			
Pre-ALB (per g)	0.078	0.990	0.980–1.001			
Dbilirubin (per *μ*mol)	0.053	1.307	0.996–1.715	0.035	1.132	1.009–1.271
Tbilirubin (per *μ*mol)	0.812	0.985	0.872–1.114			
Tcholesterol (per mmol)	0.134	1.097	0.972–1.239			

AFP: alpha fetoprotein; MVI: microvascular invasion; BCLC: Barcelona clinic liver cancer; HCV: hepatitis C virus; ALB: albumin; ALT: alanine aminotransferase; AST: aspartate aminotransferase; ALP: alkaline phosphatase; GGT: glutamyl transpeptidase; HCC: hepatocellular carcinoma.

**Table 4:** Univariate and multivariate analysis for OS in rHCC-C.

	Univariate analysis			Multivariate analysis		
	*P*	HR	95% confidence interval	*P*	HR	95% confidence interval
Gender	0.652	1.260	0.462–3.435			
Female/Male						
Age (per y)	0.206	1.018	0.990–1.048			
Length (per cm)	0.004	1.147	1.045–1.259	<0.001	1.145	1.061–1.236
Number	0.114	1.879	0.859–4.112			
Multiple/Single						
MVI	0.661	1.200	0.531–2.716			
Yes/No						
BCLC	0.077	1.443	0.961–2.168	<0.001	1.515	1.188–1.932
C/B/A						
Edmondson	0.507	1.133	0.784–1.638			
IV/III/II/I						
AFP	0.253	1.430	0.774–2.639			
>92 ng/ml/≤92 ng/ml						
HBV-DNA (per 1)	0.546	1.000	1.000–1.000			
Necrosis	0.019	0.426	0.209–0.868			
No/Yes						
Local invasion	0.128	1.662	0.864–3.199			
Yes/No						
HbsAg	0.002	0.034	0.004–0.293	0.006	0.103	0.020–0.529
No/Yes						
HCV	0.018	0.145	0.029–0.722	0.024	0.182	0.042–0.795
No/Yes						
Alcohol history	0.848	1.077	0.504–2.302			
No/Yes						
ALB (per g)	0.120	1.060	0.985–1.141			
ALT (per U)	0.315	0.995	0.985–1.005	.		
AST (per U)	0.230	1.004	0.998–1.009			
ALP (per U)	0.255	1.002	0.999–1.005			
GGT (per U)	0.071	0.996	0.992–1.000			
Creatinine (per *μ*mol)	0.039	0.972	0.947–0.999	0.007	0.976	0.958–0.993
Pre-ALB (per g)	0.349	0.996	0.987–1.005			
Dbilirubin (per *μ*mol)	0.244	0.883	0.716–1.089			
Tbilirubin (per *μ*mol)	0.085	1.085	0.989–1.190	0.017	1.031	1.005–1.058
Tcholesterol (per mmol)	0.934	0.986	0.710–1.370			

AFP: alpha fetoprotein; MVI: microvascular invasion; BCLC: Barcelona clinic liver cancer; HCV: hepatitis C virus; ALB: albumin; ALT: alanine aminotransferase; AST: aspartate aminotransferase; ALP: alkaline phosphatase; GGT: glutamyl transpeptidase; HCC: hepatocellular carcinoma.

**Table 5:** Univariate and multivariate analysis for OS in rHCC-NC.

	Univariate analysis			Multivariate analysis		
	*P*	HR	95% confidence interval	*P*	HR	95% confidence interval
Gender	0.651	1.422	0.309–6.536			
Female/Male						
Age (per y)	0.761	1.006	0.967–1.047			
Length (per cm)	0.577	1.041	0.904–1.198			
Number	0.604	1.358	0.427–4.316			
Multiple/Single						
MVI	0.442	1.786	0.407–7.843			
Yes/No						
BCLC	0.208	1.632	0.761–3.499			
C/B/A						
Edmondson	0.448	0.843	0.542–1.310			
IV/III/II/I						
AFP	0.014	3.306	1.269–8.609	0.034	1.724	1.041–2.855
>92 ng/ml/≤92 ng/ml						
HBV-DNA (per 1)	0.798	1.000	1.000–1.000			
Necrosis	0.725	0.817	0.264–2.524			
No/Yes						
Local invasion	0.775	1.148	0.447–2.949			
Yes/No						
HbsAg	0.132	2.213	0.787–6.223			
No/Yes						
HCV	0.418	2.029	0.366–11.235			
No/Yes						
Alcohol history	0.994	1.004	0.346–2.911			
No/Yes						
ALB (per g)	0.145	0.954	0.895–1.016			
ALT (per U)	0.026	0.974	0.952–0.997			
AST (per U)	0.006	1.019	1.005–1.033			
ALP (per U)	0.873	0.998	0.979–1.018			
GGT (per U)	0.504	0.995	0.979–1.010			
Creatinine (per *μ*mol)	0.529	0.990	0.959–1.022			
Pre-ALB (per g)	0.164	1.006	0.997–1.015			
Dbilirubin (per *μ*mol)	0.111	1.211	0.957–1.532			
Tbilirubin (per *μ*mol)	0.522	0.967	0.871–1.073			
Tcholesterol (per mmol)	0.715	0.972	0.836–1.131			

AFP: alpha fetoprotein; MVI: microvascular invasion; BCLC: Barcelona clinic liver cancer; HCV: hepatitis C virus; ALB: albumin; ALT: alanine aminotransferase; AST: aspartate aminotransferase; ALP: alkaline phosphatase; GGT: glutamyl transpeptidase; HCC: hepatocellular carcinoma.

## Data Availability

The datasets used and analyzed during the current study are available from the corresponding author on reasonable request.

## Abbreviations

HCC: Hepatocellular carcinoma
rHCC: Ruptured hepatocellular carcinoma
rHCC-C: Ruptured hepatocellular carcinoma with cirrhosis
rHCC-NC: Ruptured hepatocellular carcinoma without cirrhosis
HbsAg: Hepatitis B surface antigen
AFP: Alpha-fetoprotein
MVI: Microvascular invasion
BCLC: Barcelona clinic liver cancer
HCV: Hepatitis C virus
ALB: Albumin
ALT: Alanine aminotransferase
AST: Aspartate aminotransferase
ALP: Alkaline phosphatase
GGT: Glutamyl transpeptidase
DCA: Decision curve analysis
CT: Computed tomography
MRI: Magnetic resonance imaging
RFA: Radiofrequency ablation
TACE: Transcatheter arterial chemoembolization
OS: Overall survival
RFS: Recurrence-free survival.
